# Optimization of Gentisides Extraction from *Gentiana rigescens* Franch. ex Hemsl. by Response Surface Methodology

**DOI:** 10.1155/2015/819067

**Published:** 2015-12-24

**Authors:** Bowen Chu, Yao Shi, Zhimin Li, Hao Tian, Wanyi Li, Yuanzhong Wang

**Affiliations:** ^1^Institute of Medicinal Plants, Yunnan Academy of Agricultural Sciences, Kunming 650200, China; ^2^College of Traditional Chinese Medicine, Yunnan College of Traditional Chinese Medicine, Kunming 650500, China; ^3^Yunnan Technical Center for Quality of Chinese Materia Medica, Kunming 650200, China

## Abstract

Gentisides are a class of chemical compounds which is considered as potential therapeutic substance for treatment of neurodegenerative disorders. The heat reflux extraction conditions were optimized for seven kinds of gentisides from the root and rhizome of* Gentiana rigescens* Franch. ex Hemsl. by employing response surface method. Based on univariate test, a Box-Behnken design (BBD) was applied to the survey of relationships between response value (gentisides yield) and independent variables which were chosen from various extraction processes, including extraction temperature, extraction time, and solvent-material ratio. The optimized conditions for this extraction are as follows: extraction time of 3.40 h, extraction temperature of 74.33°C, and ratio of solvent to raw material of 10.21 : 1 mL/g. Verification assay revealed that the predicted value (99.24%) of extraction parameters from this model was mainly conformed to the experimentally observed values (98.61 ± 0.61).

## 1. Introduction

*Gentiana* (family: Gentianaceae) was known as an important traditional Chinese medicinal (TCM) herb, namely, called “Long-Dan” in Chinese, whose various bioactivities contain hepatoprotective, anti-inflammatory, analgesic, antiproliferative, and antimicrobial effects. Iridoid and secoiridoid, such as loganic acid, gentiopicroside, sweroside, and swertiamarin were considered as main constituents in four* Gentiana* species recorded as* G. manshurica* Kitag. (Tiao-Ye-Long-Dan),* G. scabra* Bge. (Cu-Cao-Long-Dan, Long-Dan),* G. triflora* Pall. (San-Hua-Long-Dan), and* G. rigescens* Franch. ex Hemsl. (Dian-Long-Dan), respectively [1, 2].

**Figure 1:**
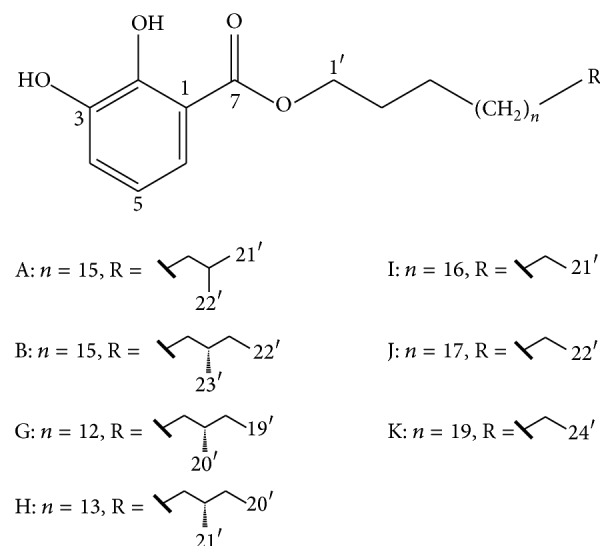
Chemical constitution of seven kinds of gentisides (gentisides A, B, G, H, I, J, and K).

Nerve growth factor (NGF) is one of the most important neurotrophic factors to act on the efficacity such as neuronal differentiation, growth, survival, function maintenance, and prevention of aging in the central and peripheral systems [3, 4]. However, previous investigations revealed that neuritogenic agent is limited to use because that NGF cannot pass through the blood-brain barrier due to its large molecular size and hydrophilic [5]. Gentiside is a kind of new neuritogenic activity compounds (shown in Figure 1) isolated from* G. rigescens*, which was researched to enhance the activity of endogenous neurotrophic factors and exhibited neurite elongation in PC_12_ cells and gentisides' bioactivity was related to only alkyl chain length, not structural diversity of end of the alkyl chain [6–8]. Chemical structures of gentisides (A, B, G, H, I, J, and K) had been concluded in Figure 1. Sequentially, Luo et al. [9] summarized structure-activity relationships of a series of synthesized gentiside derivatives and implied that the class of compound was promising agents for the treatment of neurodegenerative diseases. Alzheimer's disease (AD) is most commonly causing dementia on a world scale and much more significantly influencing normal daily life than other diseases [5, 10]. The development of using gentisides as a therapeutic drug for neurological impairment could properly replace NGF which was once thought of as most potential AD drug [11]. Thus, gentisides possess great significance and development value in neurodegenerative disorders sphere.

Response surface methodology (RSM) is a common tool to research the development of an adequate functional relationship between a response of interest (*Y*) and a number of associated control (or input) variables, which consists of a group of mathematical and statistical techniques [12]. Compared with traditional optimization method, major advantage of RSM is that it can perform the interactive effects among the studied variables. Full three-level factorial, Box-Behnken, central composite, and Doehlert designs were used most frequently for RSM of experimental design [13]. Compared with central composite and three-level full factorial design, the Box-Behnken design employed in this experiment and Doehlert matrix design demonstrated slightly more efficiency than others [14]. Previous researches about gentisides were progressive and continuous; however, the knowledge of extraction process about the active constituent has not been described. Thus, we attempted to seek optimum conditions for gentisides extraction according to RSM.

## 2. Materials and Methods

### 2.1. Materials and Reagents

The root and rhizome of* G. rigescens* collected from Yunxian (Lincang City, Yunnan Province, China) were identified by Professor Hang Jin from Yunnan Academy of Agricultural Science (YAAS). The herbariums were preserved in the Institute of Medicinal Plants, YAAS. The material specimens dried at 50°C in oven for a week were ground through a 60-mesh sieve and then utilized as test spares. HPLC grade methanol and acetonitrile (Sigma Inc., USA) were used as eluents A and B for chromatographic analysis. All the other reagents including ethanol, trichloromethane, and cyclohexane were used in analytical grade.

### 2.2. Heat Reflux Extraction

Samples of 2 g were extracted by means of conventional heat reflux extraction using 90% ethanol solvent in a given extraction temperature, extraction time, and solvent-material ratio. The above operations repeated twice. The combined hot extracts of samples were collected and filtered through filter paper. A process of decompressing concentration was implemented with the filtration liquid using a rotary evaporator (BÜICH, Flawil, Switzerland) at 45°C until becoming highly enriched extractum.

### 2.3. Purification Process of Primary Extract

The previous extractive liquor was loaded into SPE (Solid Phase Extraction) silica gel column chromatography (500 mg/3 mL, WondaSep, SHIMADZU-GL, Tokyo, Japan). Eluents C and D (ratios of cyclohexane to chloroform were 9 : 1 and 7 : 3, resp.) were used for eluting the active ingredients from the silica gel column, and usage amount was 1.5 mL and 5 mL, respectively. Elution fractions of eluent D were collected and concentrated to dry and then dissolved by ethanol absolute of 1 mL for subsequent determination by HPLC [15].

### 2.4. Determination and Analysis of Gentisides by HPLC

Chromatographic analysis was carried out on a Dionex U3000 HPLC system (Dionex, Sunnyvale, USA) equipped with DAD detector. A reversed-phase WondaCract ODS-2 column (5 *μ*m, 4.6 mm × 250 mm; SHIMADZU-GL, Japan) was used for separating the purified samples. The mobile phase consisted of methanol as eluent A and acetonitrile as eluent B which was performed with flow rate of 1.0 mL/min for one hour. The ratio of eluent A to eluent B was 4 : 6, column temperature was 30°C, and the injection volume was 20 *μ*L. UV detection wavelength of the monitor was arranged at 210 nm [15]. Data processing was carried out with Chromeleon 6.80 SR9a software.

Content of gentisides could be calculated by establishing standard curve. The gentisides yield (GY) was calculated using the following equation:

(1) gentisides yield%=∑i=1n=7YiY0,Y1−7=YA,YB,YG,YH,YI,YJ,YK,

where *Y*_*i*_ represented the value of gentisides content calculated by corresponding peak area sourced from HPLC and *Y*_0_ was estimated value of sample, which was evaluated as 3 mg previously.

### 2.5. Univariate Test Design

Concentration of extraction solution (ethanol-water solution), extraction temperature, extraction time, and solvent-material ratio were considered as main influence factors to gentisides yield extraction. The parameters and their ranges were as follows: concentration of extracting solution of 60–90%, extraction temperature of 55–85°C, extraction time of 0.5–4 h, and ratio of liquid to material of 1 : 4–1 : 12 (g/mL).

### 2.6. Optimum Experiment Design and Statistical Analysis

The parameters of extraction conditions were optimized using response surface methodology. Based on the consequence of univariate test, a BBD (three factors, three levels) experiment was performed to elucidate the relationships between independent variables (*X*_1_, *X*_2_, and *X*_3_), and exclusive response value (*Y*) [13]. The actual values were coded at three levels: −1, 0, and +1. Experimental design scheme derived from Design-Expert 8.0.6 software (Stat-Ease Inc., MN, USA) and response value (gentisides yield) were shown in Table 1. The four variables were coded according to the following equation:

(2) $$\begin{array}{c} X_{i}=\frac{x_{i}-x_{0}}{\Delta x}, \end{array}$$

where *X*_*i*_ is the coded value, *x*_*i*_ is the corresponding actual value, *x*_0_ is the actual value in the center of the domain, and Δ*x* is the increment of *x*_*i*_ corresponding to a variation of 1 unit of *X* (Supplementary Material 2 in Supplementary Material available online at http://dx.doi.org/10.1155/2015/819067). Regression coefficients for intercept, linear, quadratic, and interaction terms and nonlinear quadratic polynomial model employed were as follows:

(3) $$\begin{array}{c} Y=\beta_{0}+\overset{k}{\underset{i=0}{\sum }}\beta_{i}x_{i}+\overset{k}{\underset{j=0}{\sum }}\beta_{ii}x_{i}^{2}+\overset{k}{\underset{i=0}{\sum }}\;\overset{k}{\underset{j=0}{\sum }}\beta_{ij}x_{i}x_{j}, \end{array}$$

where *Y* was the response function; *β*_0_ was a constant; *β*_*i*_, *β*_*ii*_, and *β*_*ij*_ were the linear, quadratic, and interactive coefficients, respectively; and *x*_*i*_ represented the coded levels of independent variables. The terms *x*_*i*_ *x*_*j*_ and *x*_*i*_^2^ were expressed as the interaction and quadratic terms, respectively.

## 3. Results and Discussion

### 3.1. Chromatographic Results

**Figure 2:**
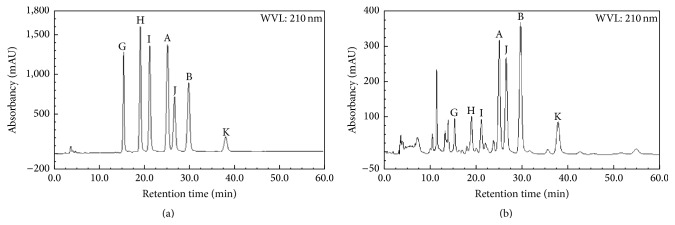
The HPLC chromatograms of gentisides A, B, G, H, I, J, and K; (a) the chromatogram of standard substance; (b) the chromatogram of sample.

The HPLC chromatograms of standard substance and testing sample were shown in Figures 2(a) and 2(b). Absorption peaks of the target compounds were arisen at retention time of 15.180, 18.917, 21.040, 25.047, 26.610, and 29.783 min. The calibration curves were plotted by using five gradient concentrations of mixed standard substance (0.01, 0.05, 0.10, 0.50, and 1.00 mg/mL), which quantified the GY (expressed as %) by means of regressive equations: *Y* = 735.88*x* + 0.9700 (gentiside A, *R* ^2^ = 0.9994), *Y* = 446.81*x* − 2.2227 (gentiside B, *R* ^2^ = 0.9976), *Y* = 419.66*x* − 0.1712 (gentiside G, *R* ^2^ = 0.9988), *Y* = 655.49*x* + 2.2037 (gentiside H, *R* ^2^ = 0.9989), *Y* = 630.77*x* + 1.2064 (gentiside I, *R* ^2^ = 0.9987), *Y* = 417.80*x* + 2.1768 (gentiside J, *R* ^2^ = 0.9985), and *Y* = 130.69*x* − 0.1592 (gentiside K, *R* ^2^ = 0.9995), where *Y* denoted peak area and *x* denoted concentration of sample (linear intervals were shown in Supplementary Material 3). HPLC conditions including precision, stability, and repeatability were summarized in Supplementary Material 4, which revealed that the experiment conditions performed well enough to carry on sample analysis.

### 3.2. Effect of the Ethanol Concentration on the Gentisides Yield

**Figure 3:**
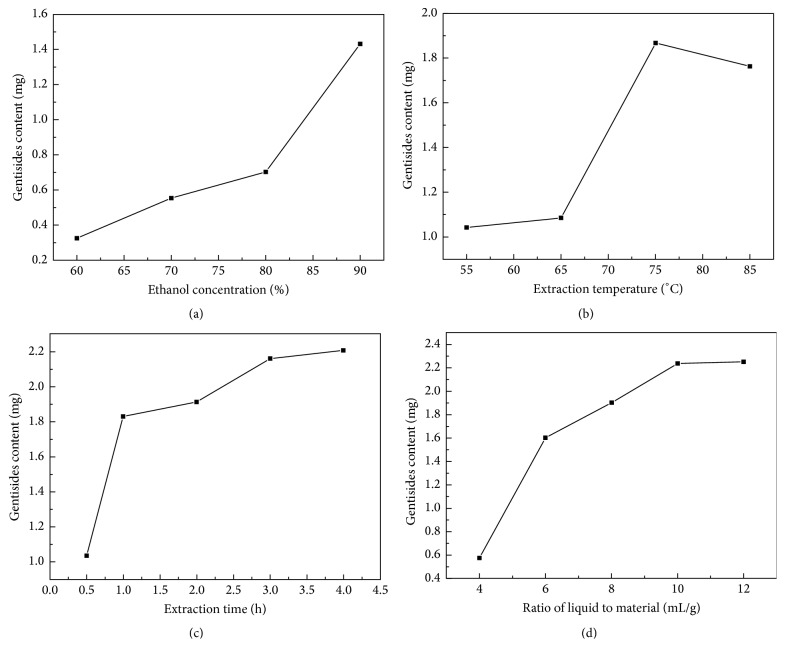
Experimental results of univariate test in four kinds of extraction conditions; (a) effect of the ethanol concentration selected as 60%, 70%, 80%, and 90%; (b) effect of the extraction temperature set at 55, 65, 75, and 85°C; (c) effect of the extraction time set at 0.5, 1, 2, 3, and 4 h; (d) effect of the ratio of liquid to material selected as 4, 6, 8, 10, and 12 mL/g.

We selected, respectively, 60%, 70%, 80%, and 90% as the concentration of ethanol-water to investigate the effect of discrepant concentration of the extraction solvent on the gentisides yield when other extraction parameters were as follows: extraction temperature 75°C, extraction time of 3 h, and solvent-material ratio of 10 mL/g. As shown in Figure 3(a), the content of gentisides was rising straight along with increasing concentration of ethanol and finally we obtained the highest content of gentisides in concentration of 90%. Therefore, we selected 90% as concentration of solvent in this experiment.

### 3.3. Effect of the Extraction Temperature on the Gentisides Yield

The extraction temperature was set at 55, 65, 75, and 85°C, respectively, to examine the effect of different temperature on the gentisides yield when other extraction parameters were as follows: ethanol-water concentration of 90%, extraction time of 3 h, and solvent-material ratio of 10 mL/g. As shown in Figure 3(b), a distinct increase of the content of gentisides emerged in plate from 55 to 75°C, and then the content of gentisides dropped slightly from 75 to 85°C. It could be explained that ester groups of effective components were resolved at high temperature [16, 17]. The statistical analysis showed that significant differences existed among 55, 65, 85, and 75°C, and the content of gentisides reached the highest temperature at 75°C. As a result, we selected 75°C as the center point of extraction temperature in this experiment.

### 3.4. Effect of the Extraction Time on the Gentisides Yield

The extraction time was set at 0.5, 1, 2, 3, and 4 h, respectively, to examine the effect of different extraction time on the gentisides yield when other extraction parameters were as follows: ethanol-water concentration of 90%, extraction temperature of 75°C, and solvent-material ratio of 10 mL/g. As shown in Figure 3(c), the content of gentisides was rising straight as extraction time from 0.5 to 1 h and then rose up slightly from 1 to 4 h. As a result, we selected 2 h as the center point of extraction time in this experiment.

### 3.5. The Effect of the Ratio of Liquid to Material on the Gentisides Yield

Respectively, 4, 6, 8, 10, and 12 mL/g were selected as the ratio of liquid to material of extraction parameter to investigate the effect of different extraction time on the gentisides yield when other extraction parameters were as follows: ethanol-water concentration of 90%, extraction temperature of 75°C, and solvent-material ratio of 10 mL/g. As shown in Figure 3(d), the content of gentisides was rising straight as solvent-material ratio rising from 4 to 10 mL/g, particularly at the beginning of solvent volume increase, and then rose up inconspicuously after 10 mL/g. To maximize gentiside yield, we selected 10 mL/g as the center point of solvent-material ratio in this experiment.

### 3.6. Optimization Parameters by Response Surface Method

The extraction of gentisides from* Gentiana rigescens* was optimized through response surface methodology. An optimum result could be obtained from optimized model according to statistical analysis. All the 17 of the designed experiments were disorderedly conducted (for the decrease of uncontrollable influence) on the basis of Table 1. Response variable *Y* predicted and the test variables (*X*_1_, *X*_2_, and *X*_3_) were related by the following second-order polynomial equation:

(4) Y=+98.08−0.49X1+5.26X2+1.82X3−7.02X1X2−2.55X1X3−3.77X2X3−26.40X12−6.71X22−2.35X32,

where *Y* is function of the regression equation, namely, GY (%), and *X*_1_, *X*_2_, and *X*_3_ are the coded variables for extraction temperature, extraction time, and the ratio of solvent to the raw material, respectively.

As shown in Table 2, the analysis of variance (ANOVA) was employed for statistical significance of response surface quadratic polynomial model. Here, model *F*-value of 12.68 (more than *F*-critical value of 8.81) and model *P* value (95% confidence level) of 0.0015 (<0.01) indicated that the quadratic polynomial model was significant in order to suitably predict optimal experiment conditions. The lack of fit measures the failure of the model to represent the data in the experimental domain at points which are not included in the regression [18]. As listed in Table 2, *F*-value and *P* value of the lack of fit were 0.39 and 0.7674, respectively, which indicated that it was not significant relative to the pure error and implied that the model equation was available. The higher the value of *R*_adj_^2^ is, the deeper the correlation between the observed and predicted values performs [19]. The value of *R* ^2^ (determination coefficient) was 0.9422, which indicated the satisfactory correlation between actual values and predicted ones [20, 21]. The value of *R*_adj_^2^ (adjusted determination coefficient) was 0.8679, which meant that at least 86.79% of the gentisides content could be predicted by the model, and only less than 1.3% of the total variations were not explained by model. Adequate precision compares the range of the predicted values at the design points to the average prediction error. A ratio greater than 4 indicated adequate model discrimination. As shown in Table 2, the value of adequate precision was 10.122, which indicated an adequate signal for model and could be used to navigate the design space. CV (coefficient of variation) indicates the degree of precision with which the experiments are compared. As shown in Table 2, a relatively low value of CV was 1.14, which showed a better precision and reliability of quadratic polynomial model [18].

The *P* value is used as a tool to check the significance of each coefficient and the interaction strength between each independent variable [22]. According to the *P* values of each model term summarized in Table 2, it could be concluded that these terms including an independent variable (*X*_2_) and two quadratic terms (*X*_1_^2^ and *X*_2_^2^) were significantly affecting the yield of gentisides (“Prob > *F*” less than 0.05), and among them quadratic terms were the most significant parameters which influenced GY. It also shown that interaction between extraction temperature (*X*_1_) and extraction time (*X*_2_) was significant. Thus, the extraction temperature (*X*_1_) was confirmed as the key condition affecting the GY.

### 3.7. Analysis of the Response Surface

**Figure 4:**
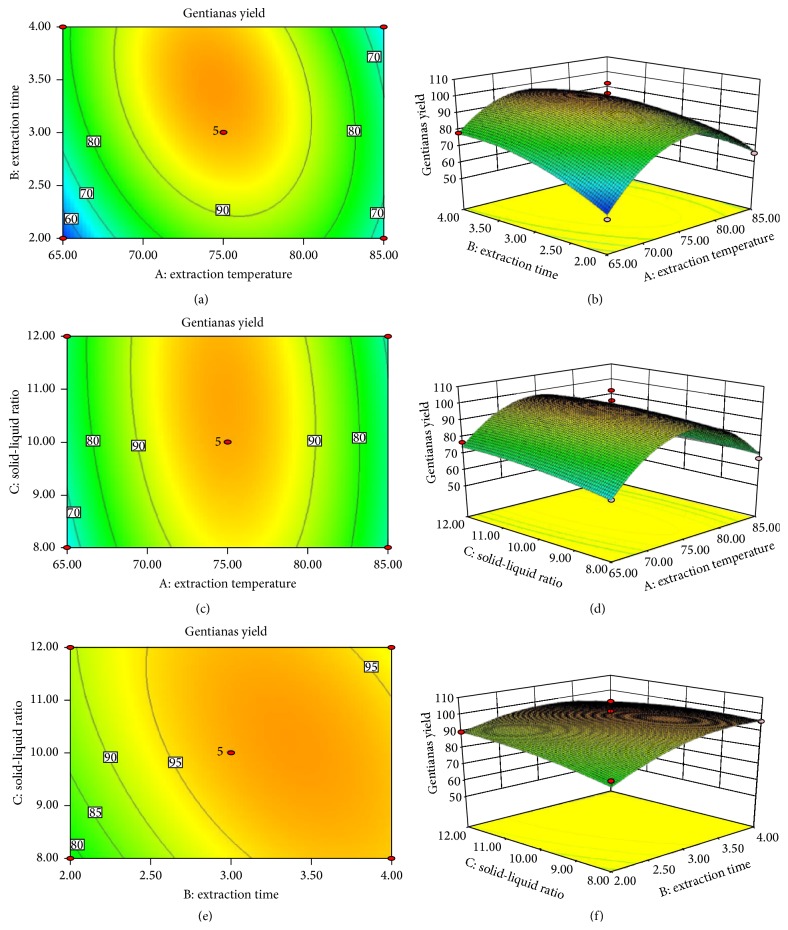
2D contour plots and 3D response surface plots of the effects on relationships between independent variables and response value; ((a) and (b)) 2D contour and 3D plot of the interactions between extraction time and extraction temperature; ((c) and (d)) 2D contour and 3D plot of the interactions between extraction temperature and the ratio of liquid to material; ((e) and (f)) 2D contour and 3D plot of the interactions between extraction time and the ratio of liquid to material.

The 2D contour plots and 3D response surface plots graphically represented the interactive effects of extraction temperature (*X*_1_), extraction time (*X*_2_), and solvent-material ratio (*X*_3_) on GY. According to contour plots' shapes produced by Design-Expert 8.0.6., response surface plots demonstrated whether the mutual interactions between the variables are significant or not, which were shown in Figure 4. Circular contour plot indicates that the interactions between the corresponding variables are negligible, while elliptical contour plot indicates that the interactions between the corresponding variables are significant [22]. As two variables were depicted in same plots while the other variable was kept at level 0, relationships between independent and dependent variables could be intuitively conveyed. Figures 4(b) and 4(d) showed the interaction relationships of extraction temperature (*X*_1_) with the extraction time (*X*_2_) and solvent-material ratio (*X*_3_) on the yield of gentisides, respectively. The yield of gentisides increased rapidly with the increment of extraction temperature, while it declined rapidly with higher temperature after a critical value of 75°C. The yield of gentisides increased lightly along with extraction time extended (from 2 to 4 h) at extraction temperature lower than 80°C and even declined less obviously at extraction temperature higher than 80°C, which indicated that mutual interaction of extraction temperature and extraction time was significant on the increasing of the yields extraction. The response curves of extract variable *X*_3_ in Figure 4(d) were smooth at all degree extraction temperatures indicating that solvent-material ratio was insignificant on the increasing of the yields extraction. In Figure 4(f), the response surface was smooth, which expressed that the mutual interaction of extraction time and solvent-material ratio had unconspicuous significance on the extraction yield. Descriptions above were in general accord with data (*P* < 0.05) in Table 2.

### 3.8. Optimization of Extracting Parameters and Validation of the Model

A series of experiments were performed to obtain optimum conditions for independent variables and the predicted values of the responses by using Design-Expert 8.0.6 software. Optimal values of extraction parameters and maximum predicted yield of gentisides were given in Table 3 and also carried out as follows: extraction temperature of 74.33°C, extraction time of 3.40 h, solvent-material ratio of 10.21 mL/g, solvent concentration (ethanol) of 90%, and optimum predicted value of response of 99.24%, respectively. However, considering actual production, optimized solutions were modified slightly as follows: extraction temperature of 75°C, extraction time of 3.5 h, and solvent-material ratio of 10 mL/g. Under modified conditions, actual extraction yield was 98.61 ± 0.61%, which validated that the model was adequate for predicting the maximum extraction yield of gentisides.

## 4. Conclusions

This investigation in the successful uses of univariate test and Box-Behnken design forecasted optimal extracting parameters of gentisides (gentisides A, B, G, H, I, J, and K) from the root and rhizome of* G. rigescens*, which demonstrated that maximized gentisides yield of 99.24% could be achieved when solvent concentration of 90%, extraction temperature of 74.33°C, extraction time of 3.40 h, and solvent-material ratio of 10.21 mL/g were selected as extraction conditions. Considering feasible technical operations, solvent concentration of 90%, extraction temperature of 75°C, extraction time of 3.5 h, and solvent-material ratio of 10 mL/g were determined as final optimum extraction conditions.

## Supplementary Material

Supplementary Material 1: Gentisides were series of new neuritogenic activity compounds isolated from *G. rigescens*, and their bioactivity was related to only alkyl chain length, not structural diversity of end of the alkyl chain. Chemical structural formula of seven kinds of gentiside, namely gentiside A, gentiside B, gentiside G, gentiside H, gentiside I, gentiside J, and gentiside K, had been drawn as following.
Supplementary Material 2: Based on the univariate test, extraction temperature, extraction time and ratio of liquid to material were selected as three-factors in BBD experiment. And three level points in investigated range were respectively coded as -1, 0 and +1 for the sake of experimental design.
Supplementary Material 3: According to calculating by seven various concentrations, seven created regression equations performed a good linear relationship in given linear ranges and the correlation coefficients (R2) were ≥0.99 for all of the compounds over corresponding linearity range.
Supplementary Material 4: To ensure our experimental equipment and data to be reliable, HPLC instrument received a validation including precision, stability, repeatability and recovery in this work. In order to assess the intra-day and inter-day precision of the method, standard solution was analyzed in a day (0, 2, 4, 8, 16 and 24 h) and three consecutive days (0, 24, 36, 48, 60 and 72 h), respectively. To test the stability of samples, unitary extract solution was analyzed by HPLC within a day (0, 2, 4, 8, 16 and 24 h). To confirm the repeatability, six repeated samples were once test. The RSD (%) of each item less than 3 meant the described method had an accepted degree of precision.

## Figures and Tables

**Table 1:** Box-Behnken design and the actual and coded values of the independent variables.

Run	Extraction temperature	Extraction time	Solvent-material ratio	Gentisides yield
	*X*_1_: °C	*X*_2_: h	*X*_3_: mL/g	*Y*: (%)
1	0 (75)	0 (3)	0 (10)	95.7591
2	0 (75)	−1 (2)	−1 (8)	81.2493
3	0 (75)	0 (3)	0 (10)	101.9360
4	−1 (65)	0 (3)	−1 (8)	65.4543
5	−1 (65)	0 (3)	+1 (12)	76.8498
6	0 (75)	0 (3)	0 (10)	92.8269
7	0 (75)	−1 (2)	+1 (12)	89.7775
8	0 (75)	0 (3)	0 (10)	91.9897
9	+1 (85)	+1 (4)	0 (10)	65.7978
10	0 (75)	+1 (4)	+1 (12)	89.2336
11	0 (75)	+1 (4)	−1 (8)	95.8044
12	0 (75)	0 (3)	0 (10)	107.89
13	+1 (85)	0 (3)	+1 (12)	68.1115
14	+1 (85)	−1 (2)	0 (10)	65.8044
15	−1 (65)	−1 (2)	0 (10)	50.0952
16	+1 (85)	0 (3)	−1 (8)	66.9001
17	−1 (65)	+1 (4)	0 (10)	78.1553

**Table 2:** Analysis of variance for response surface quadratic polynomial model. SD: sources of deviation; SS: sum of squares; DF: degree of freedom; MS: mean square; *R* ^2^: determination coefficient; *R*_adj_^2^: adjusted determination coefficient; CV %: coefficient of variation.

SD	SS	DF	MS	*F* value	*P* value
Model	3816.19	9	424.02	12.68	0.0015
*X*_1_	1.94	1	1.94	0.06	0.8165
*X*_2_	221.18	1	221.18	6.61	0.0369
*X*_3_	26.51	1	26.51	0.79	0.4029
*X*_1_ *X*_2_	196.93	1	196.93	5.89	0.0457
*X*_1_ *X*_3_	25.93	1	25.93	0.78	0.4078
*X*_2_ *X*_3_	56.99	1	56.99	1.70	0.2331
*X*_1_^2^	2935.05	1	2935.05	87.74	<0.0001
*X*_2_^2^	189.85	1	189.85	5.68	0.0487
*X*_3_^2^	23.24	1	23.24	0.69	0.4321
Residual	234.16	7	33.45		
Lack of fit	52.99	3	17.66	0.39	0.7674
Pure error	181.17	4	45.29		
Cor. total	4050.35	16			

*R* ^2^	0.9422		Adeq. precision	10.122	
*R*_adj_^2^	0.8679		C.V. %	7.11	
Pred. *R* ^2^	0.7208		PRESS	1130.88	

**Table 3:** Optimum conditions and predicted and experimental values of the responses at actual extraction conditions.

	Extraction temperature (°C)	Extraction time (h)	Solvent-material ratio (mL/g)	Yield of gentisides (%)
Optimum conditions	74.33	3.4	10.21	99.24 (predicted)
Modified conditions	75	3.5	10	98.61 ± 0.61 (actual)

## References

[1] Chinese Pharmacopoeia Commission (2015). *Pharmacopoeia of the People's Republic of China (Part One)*.

[2] Wang Y. M., Xu M., Wang D., Zhu H., Yang C., Zhang Y. (2012). Review on ‘*Long-Dan*’, one of the traditional Chinese medicinal herbs recorded in Chinese pharmacopoeia. *Natural Products and Bioprospecting*.

[3] Levi-Montalcini R. (1987). The nerve growth factor 35 years later. *Bioscience Reports*.

[4] Kromer L. F. (1987). Nerve growth factor treatment after brain injury prevents neuronal death. *Science*.

[5] Brinton R. D., Yamazaki R. S. (1998). Advances and challenges in the prevention and treatment of Alzheimer's disease. *Pharmaceutical Research*.

[6] Gao L. J., Li J., Qi J. (2010). Gentisides A and B, two new neuritogenic compounds from the traditional Chinese medicine *Gentiana rigescens* Franch. *Bioorganic & Medicinal Chemistry*.

[7] Gao L. J., Xiang L., Luo Y., Wang G., Li J., Qi J. (2010). Gentisides C–K: nine new neuritogenic compounds from the traditional Chinese medicine *Gentiana rigescens* Franch. *Bioorganic & Medicinal Chemistry*.

[8] More S. V., Koppula S., Kim I.-S., Kumar H., Kim B.-W., Choi D.-K. (2012). The role of bioactive compounds on the promotion of neurite outgrowth. *Molecules*.

[9] Luo Y., Sun K. Y., Li L. (2011). Structure-activity relationships of neuritogenic gentiside derivatives. *ChemMedChem*.

[10] Henderson V. W. (1997). Estrogen replacement therapy for the prevention and treatment of Alzheimer's disease. *CNS Drugs*.

[11] Hefti F. (1994). Development of effective therapy for Alzheimer's disease based on neurotrophic factors. *Neurobiology of Aging*.

[12] Khuri A. I., Mukhopadhyay S. (2010). Response surface methodology. *Wiley Interdisciplinary Reviews: Computational Statistics*.

[13] Bezerra M. A., Santelli R. E., Oliveira E. P., Villar L. S., Escaleira L. A. (2008). Response surface methodology (RSM) as a tool for optimization in analytical chemistry. *Talanta*.

[14] Ferreira S. L. C., Bruns R. E., Ferreira H. S. (2007). Box-Behnken design: an alternative for the optimization of analytical methods. *Analytica Chimica Acta*.

[15] Shi Y., Tian H., Li W. Y. (2015). Simultaneous determination of seven benzoate compounds from *Gentiana rigescens* Franch. by solid phase extraction and HPLC. *Chinese Journal of Chemical Analysis Laboratory*.

[16] Hiskey M. A., Brower K. R., Oxley J. C. (1991). Thermal decomposition of nitrate esters. *Journal of Physical Chemistry*.

[17] Ramirez M. L., Walters R., Lyon R. E., Savitski E. P. (2002). Thermal decomposition of cyanate ester resins. *Polymer Degradation and Stability*.

[18] Zhong K., Wang Q. (2010). Optimization of ultrasonic extraction of polysaccharides from dried longan pulp using response surface methodology. *Carbohydrate Polymers*.

[19] Ravilumar K., Ramalingam S., Krishnan S., Balu K. (2006). Application of response surface methodology to optimize the process variables for reactive red and acid brown dye removal using a novel adsorbent. *Dyes and Pigments*.

[20] Silva E. M., Rogez H., Larondelle Y. (2007). Optimization of extraction of phenolics from *Inga edulis* leaves using response surface methodology. *Separation and Purification Technology*.

[21] Sinha K., Saha P. D., Datta S. (2012). Response surface optimization and artificial neural network modeling of microwave assisted natural dye extraction from pomegranate rind. *Industrial Crops and Products*.

[22] Muralidhar R. V., Chirumamilla R. R., Ramachandran V. N., Marchant R., Nigam P. (2001). Racemic resolution of RS-baclofen using lipase from *Candida cylindracea*. *Mededelingen*.

