# Supplementary material for: Optimization of Gentisides Extraction from Gentiana rigescens Franch. ex Hemsl. by Response Surface Methodology
Source: J Anal Methods Chem. 2015 Dec 24;2015:819067. doi: 10.1155/2015/819067 (PMC4707016; doi:10.1155/2015/819067)
Supplement: Supplementary file 1 — Supplementary Material 1: Gentisides were series of new neuritogenic activity compounds isolated from G. rigescens, and their bioactivity was related to only alkyl chain length, not structural diversity of end of the alkyl chain. Chemical structural formula of seven kinds of gentiside, namely gentiside A, gentiside B, gentiside G, gentiside H, gentiside I, gentiside J, and gentiside K, had been drawn as following. Supplementary Material 2: Based on the univariate test, extraction temperature, extraction time and ratio of liquid to material were selected as three-factors in BBD experiment. And three level points in investigated range were respectively coded as -1, 0 and +1 for the sake of experimental design. Supplementary Material 3: According to calculating by seven various concentrations, seven created regression equations performed a good linear relationship in given linear ranges and the correlation coefficients (R2) were ≥0.99 for all of the compounds over corresponding linearity range. Supplementary Material 4: To ensure our experimental equipment and data to be reliable, HPLC instrument received a validation including precision, stability, repeatability and recovery in this work. In order to assess the intra-day and inter-day precision of the method, standard solution was analyzed in a day (0, 2, 4, 8, 16 and 24 h) and three consecutive days (0, 24, 36, 48, 60 and 72 h), respectively. To test the stability of samples, unitary extract solution was analyzed by HPLC within a day (0, 2, 4, 8, 16 and 24 h). To confirm the repeatability, six repeated samples were once test. The RSD (%) of each item less than 3 meant the described method had an accepted degree of precision. [file 819067.f1.pdf]

Supplementary Material

Supplementary Material 1: Structures of gentisides A, B, G, H, I, J and K

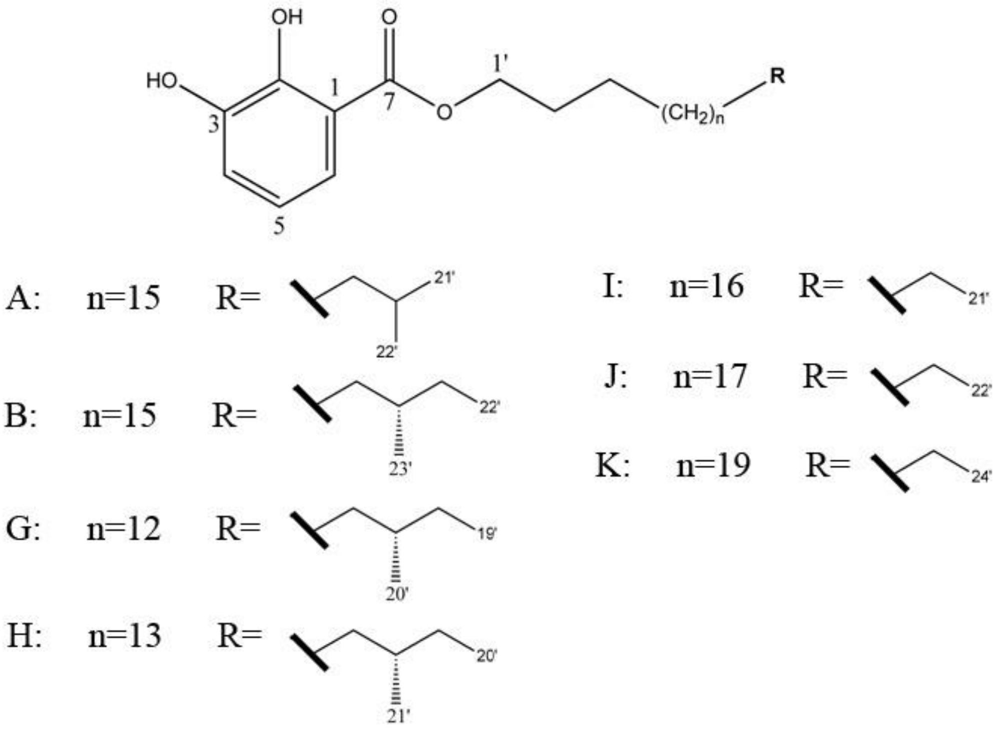

Supplementary Material 2: The actual and coded values of the independent variables

independent variables	coded values		
	-1	0	1
extraction temperature (X_1: ℃)	65	75	85
extraction time (X_2: h)	2	3	4
solvent-material ratio (X_3: mL/g)	8	10	12

Supplementary Material 3: Linearity and Precision of Gentiside A, B, G, H, I, J and K

Analytes.	Regression Equation	Correlation Coefficient (R^2)	Linear ranges (mg ·mL^{-1})
gentiside A	Y=735.88x+0.9700	0.9994	0.500-0.010
gentiside B	Y=446.81x-2.2227	0.9976	0.500-0.010
gentiside G	Y=419.66x-0.1712	0.9988	0.500-0.010
gentiside H	Y=655.49x+2.2037	0.9989	1.000-0.010
gentiside I	Y=630.77x+1.2064	0.9987	0.500-0.010
gentiside J	Y=417.80x+2.1768	0.9985	1.000-0.010
gentiside K	Y=130.69x-0.1592	0.9995	0.500-0.010

Supplementary Material 4: RSD values of precision, stability and repeatability in HPLC methodology validation experiment

Analytes.	Precisions RSD (%)		Stability RSD (%)	Reproducibility RSD (%)
	Intra-day	Inter-day		
gentiside A	0.95	1.85	2.38	2.56
gentiside B	1.21	1.93	2.57	2.17
gentiside G	0.77	1.39	1.74	1.57
gentiside H	0.99	1.64	1.99	1.70
gentiside I	1.35	2.14	2.77	2.33
gentiside J	0.57	0.93	1.07	1.49
gentiside K	2.06	2.58	2.58	2.79
